# Homing in on the hepatic scar: recent advances in cell-specific targeting of liver fibrosis

**DOI:** 10.12688/f1000research.8822.1

**Published:** 2016-07-19

**Authors:** Ross Dobie, Neil C. Henderson

**Affiliations:** 1 MRC Centre for Inflammation Research, The Queen's Medical Research Institute, University of Edinburgh, Edinburgh, UK

**Keywords:** liver fibrosis, myofibroblast biology, hepatic fibrosis, anti-fibrotic therapies

## Abstract

Despite the high prevalence of liver disease globally, there are currently no approved anti-fibrotic therapies to treat patients with liver fibrosis. A major goal in anti-fibrotic therapy is the development of drug delivery systems that allow direct targeting of the major pro-scarring cell populations within the liver (hepatic myofibroblasts) whilst not perturbing the homeostatic functions of other mesenchymal cell types present within both the liver and other organ systems. In this review we will outline some of the recent advances in our understanding of myofibroblast biology, discussing both the origin of myofibroblasts and possible myofibroblast fates during hepatic fibrosis progression and resolution. We will then discuss the various strategies currently being employed to increase the precision with which we deliver potential anti-fibrotic therapies to patients with liver fibrosis.

## Introduction

Liver disease is an increasing cause of morbidity and mortality worldwide. Recent data show that approximately 29 million people in the European Union suffer from a chronic liver condition ^1^. Regardless of aetiology, all chronic liver diseases, if left untreated, can result in hepatic fibrosis and eventually cirrhosis. This accumulation of extracellular matrix (ECM) in response to repetitive injury is part of a basic wound healing process; however, if left unchecked, increasingly severe fibrosis results in disordered tissue architecture and ultimately organ failure. Currently, there are no European Medicines Agency (EMEA)- or US Food and Drug Administration (FDA)-approved anti-fibrotic therapies available to treat patients with liver fibrosis. It is therefore imperative that we continue to gain a better understanding of the mechanisms driving hepatic fibrosis in order to facilitate and accelerate the development of new rational, highly targeted therapies.

Over the past 30 years, there has been a significant increase in our understanding of the cellular and molecular mechanisms driving liver fibrosis. It is universally accepted that myofibroblasts are the primary source of ECM proteins during hepatic fibrosis. Furthermore, rapid evolution in transgenic mouse technology has allowed the origin of the hepatic myofibroblast to be extensively investigated, with the resident hepatic stellate cell (HSC) considered to be a major source ^2, 3^. Hepatic fibrosis is also now recognised as a dynamic and potentially reversible process ^4, 5^. The highly successful antiviral therapies for hepatitis B and C have further highlighted fibrosis reversibility and the potential regenerative capacity of the human liver ^4^. This review will highlight some of the most recent advances in our understanding of hepatic fibrosis and how basic science research in this area is beginning to drive translation towards novel targeted therapies.

## Myofibroblasts: major therapeutic targets in liver fibrosis

Myofibroblasts are a key source of pathologic ECM in multiple organs and disease states, and the liver is no exception ^2, 6^. As a result, a considerable amount of research has focused on trying to delineate the origin of myofibroblasts in hepatic fibrosis, as well as deciphering the key signalling pathways responsible for myofibroblast activation, deactivation, and elimination in a bid to identify potential anti-fibrotic therapeutic targets.

**Figure 1.**
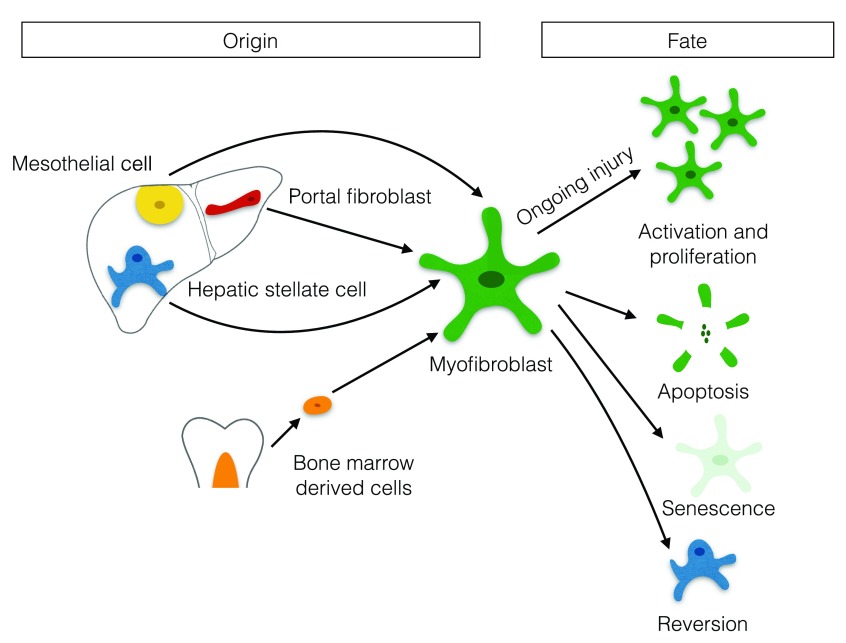
Possible origins and fates of hepatic myofibroblasts.

During hepatic fibrosis, a number of different cellular populations may contribute to the myofibroblast pool. These include HSCs, portal fibroblasts (PFs), mesothelial cells, and bone marrow-derived mesenchymal cells and fibrocytes ^7– 10^ ( Figure 1). Activation of HSCs to matrix-secreting myofibroblasts is a key process in the development of hepatic scar tissue. Recent studies have suggested that HSCs are the dominant mesenchymal contributor to the myofibroblast pool independent of the aetiology of liver disease ^7^. Using lecithin-retinol acyltransferase (Lrat)-Cre mice (labels 99% of HSCs), Mederacke and colleagues found that HSCs contribute significantly to the myofibroblast pool in models of toxic, cholestatic, and fatty liver disease ^7^. Interestingly, in studies of cholestatic liver injury, a distinct population of PF-like cells that may have a specialised function in periportal liver fibrosis were identified ^7^.

The identification and contribution of PFs to the hepatic myofibroblast pool remains less clear and is an area of active study. Given their periportal location, it is plausible that PFs may be major contributors to the myofibroblast pool associated with periportal fibrosis, and a number of studies have suggested that PFs are a major source of myofibroblasts during early cholestatic injury ^8, 11, 12^. Iwaisako *et al.* report that PFs contribute to >70% of myofibroblasts at the onset of biliary injury ^8^. This work and other studies will serve as a useful platform for the identification of PF-specific markers, facilitating the development of transgenic systems for lineage tracing and in-depth interrogation of PF function in a broad range of mouse models of liver disease. In addition, the generation of tools such as flow cytometry antibodies should allow cell-specific sorting of PFs from fibrotic human liver tissue.

More recently, a third resident mesenchymal population in the liver has attracted interest as a potential source of myofibroblasts. Li *et al.* found that following liver injury, mesothelial cells can give rise to HSCs and myofibroblasts via mesothelial to mesenchymal transition, termed MMT ^9^. Fate tracing studies using inducible Wilms Tumour 1 Cre mice (labels 14.5% of mesothelial cells) revealed that during fibrosis a number of HSCs and myofibroblasts near the liver surface derive from this subset of mesothelial cells ^9^.

In addition to studies aimed at identifying the origin of hepatic myofibroblasts and myofibroblast activation mechanisms ^13, 14^, recent work has also focused on the pathways involved in myofibroblast deactivation and elimination. Possible fates of hepatic myofibroblasts include apoptosis, senescence, and reprogramming to a deactivated state ( Figure 1) ^15– 18^. Recent data examining reprogramming of hepatic myofibroblasts found that 40–50% of myofibroblasts escape apoptosis and revert to a quiescent-like state, downregulating fibrotic genes ^15, 16^. Interestingly, both studies highlighted that reverted HSCs were distinct from quiescent HSCs and were primed, with higher responsiveness to further fibrogenic stimuli compared to quiescent HSCs ^15, 16^. The possibility of targeting and promoting myofibroblast reversion is an exciting prospect, as it could allow treatment of liver fibrosis with potentially minimal disruption to mesenchymal cell-regulated homeostasis and normal tissue repair.

## Drug targeting in the context of hepatic fibrosis

Of prime importance in the development of effective treatments for liver fibrosis, and indeed any form of organ fibrosis, is the ability to specifically target the diseased organ and pro-scarring effector cells. Therefore, over the past few years an increasing amount of research within the fibrosis arena has been aimed at the development of organ- and/or cell-specific therapy for hepatic fibrosis.

There is a long history of drug targeting to the liver, which has focused primarily on the targeting of drugs to hepatocytes. Methods include attachment of ursodeoxycholic acid (almost exclusively metabolised by hepatocytes) and asialoglycoprotein receptor-mediated targeting (primarily expressed on hepatocytes and minimally on extra-hepatic cells) ^19, 20^. Coupling of a nitric oxide-releasing derivative to ursodeoxycholic acid allows for targeted release within the liver but does not specifically target the cells primarily responsible for increased portal pressure ^19^. Other approaches have involved the use of small lipid-like nanoparticles, which are preferentially taken up by the liver. Lipid-like nanoparticles have been used successfully to deliver small interfering RNA (siRNA) against the procollagen α1 (I) gene in the liver, ultimately leading to a decrease in collagen deposition ^21^. Again, this method is not effector cell specific, as the nanoparticles are engulfed more by Kupffer cells than HSCs ^21^. Therefore, a major goal in tailored anti-fibrotic therapy involves the discovery of organ-specific and cell-specific myofibroblast markers, which would allow targeted delivery of anti-fibrotic therapies to the major pro-scarring cell population in the diseased organ.

Recently, there have been significant advances in identifying targetable markers on HSCs. Ideally, a marker would be upregulated only upon activation of HSCs to the myofibroblast phenotype. One marker that has been extensively studied in this context is platelet-derived growth factor receptor β (PDGFRβ). During hepatic fibrogenesis, HSCs transdifferentiate to myofibroblasts and upregulate PDGFRβ expression, making it an attractive candidate to allow preferential targeting of mesenchymal scar-forming cells ^13^. Harnessing PDGFRβ as a potential means of delivering anti-fibrotic treatments directly to the pool of scar-forming cells may allow a much more refined and targeted approach to the treatment of liver fibrosis. It must, however, also be considered that PDGFRβ is a broad marker of mesenchymal cells, encompassing pericytes, vascular smooth muscle cells, and resident fibroblasts in multiple organs. Therefore, the fibrosis research community must strive hard to discover myofibroblast markers and therapeutic targets that are unique to each different organ and disease state. A recent elegant study further defined which of the mesenchymal cellular populations may be responsible for myofibroblast formation and scar formation. Kramann *et al.* studied Gli1-positive cells in multiple models of organ fibrosis and found that these cells are a subset of perivascular cells committed to the myofibroblast lineage after injury ^22^. These perivascular and peribiliary Gli1-positive cells constitute only a small fraction of the PDGFRβ-positive cells in the liver but contribute to ~39% of interstitial α-smooth muscle antibody (α-SMA)-positive cells in liver fibrosis ^22^. Furthermore, Gli1-positive cells have a key role in fibrosis of other organs. Ablation of these cells reduced fibrosis by 50% in kidney and heart injury models ^22^. An informatics-based approach has also been used to identify uniquely expressed HSC genes ^23^. Included in the genes identified was protocadherin 7, which is a very promising candidate marker for HSC tracking, targeting, and isolation. Interestingly, the study also demonstrated that increased expression of HSC signature genes in patients is predictive of clinical outcomes, including decompensation, progression of Child–Pugh class, and overall survival. Combining these predictions with clinical factors led to more accurate predictions than using clinical factors alone ^23^. Ideally, anti-fibrotic therapies of the future will target the most pro-fibrotic subpopulations of the mesenchymal lineages and it is likely that identifying ‘pro-scarring’ subpopulations will become a major focus of fibrosis research in the coming years.

## Current drug delivery approaches for hepatic fibrosis

A number of different drug delivery approaches have been reported to specifically target HSCs, such as viral vectors, liposomes, and protein-based strategies ( Table 1). The first documented carrier to selectively target HSCs, mannose 6-phosphate modified albumin, was initially described over 16 years ago ^24^. Characterisation of this carrier revealed preferential binding to activated HSCs and receptor-mediated endocytosis, making it an attractive option for drug delivery ^25^. A number of studies have utilised this system to successfully target kinase inhibitors, receptor blockers, and liposomes to animal and human HSCs ^26– 28^. Targeted delivery of Rho-kinase inhibitors using this system reduced collagen deposition and lowered portal hypertension during liver fibrosis without causing the severe hypotension associated with systemic inhibition ^26, 29^. Also, short-term treatment with angiotensin type 1 receptor blockers was reported to be more efficacious in inhibiting advanced fibrosis when targeted using this carrier ^27^. Although promising, the translational potential of the mannose 6-phosphate modified albumin system may be limited by the complex processes required to synthesise these proteins, making them potentially less feasible for clinical consideration ^30^. Furthermore, long-term delivery of oligonucleotides using a mannose 6-phosphate bovine serum albumin (BSA) carrier has been associated with potential immune reactions owing to the high molecular weight of BSA ^31^. This led to the replacement of BSA with a co-polymer that negates the immunogenic response ^31^. There are reports that mannose 6-phosphate carriers are not completely HSC specific ^28, 32^. Given the negative charge associated with the mannose 6-phosphate carrier, it has been suggested that they could also be a target for scavenger receptors such as those found on Kupffer cells and endothelial cells ^28^.

**Table 1.** Current drug targeting approaches in hepatic fibrosis.

Target	Carrier +/- agent delivered
**M6P-IGF2R**	M6P-HSA (used to deliver Rho-kinase inhibitor [Y26732], angiotensin type 1 receptor blocker [losartan]) ^24, 26, 27, 29^
	M6P-BSA ^24, 25^
	M6P-HSA liposomes ^28^
	M6P-HPMA (used to deliver triplex forming oligonucleotides) ^31^
	M6P (used to deliver IL-10) ^32^
**Collagen type VI R**	pCVI-HSA ^33^
	cRGD-SSL (used to deliver IFN-α1b) ^34^
**PDGFRβ**	PPB-HSA ^35^
	BiPPB (used to deliver IFNγ peptidomimetic) ^36^
	PPB (used to deliver IFNγ) ^37^
	PDGFR-recognising peptide adenovirus ^42^
**Vitamin A storing cells**	Vitamin A liposomes (used to deliver siRNA) ^38^
**Synaptophysin R**	Single chain antibody (termed C1–3) (used to deliver gliotoxin) ^40, 41^
**p75 neurotrophin R**	p75NTRp-adenovirus (used to deliver transcription factors) ^43^
	NGFp–vector particles ^44^
**Myofibroblasts**	Adeno-associated virus vectors (used to deliver transcription factors) ^45^

Abbreviations: BiPPB, bicyclic peptide that recognises the PDGFRβ; BSA, bovine serum albumin; cRGD, cyclic peptide containing Arg-Gly-Asp; IFN, interferon; IGF2R, insulin-like growth factor 2 receptor; IL, interleukin; HPMA, N-(2-hydroxypropyl) methacrylamide copolymer; HSA, human serum albumin; M6P, mannose 6-phosphate; NGFp, nerve growth factor peptide; pCVI, cyclic peptide that recognizes the collagen type VI receptor; PDGFRβ, platelet-derived growth factor receptor β; PPB, cyclic peptide that recognises the PDGFRβ; p75NTRp, p75 neurotrophin receptor peptide; R, receptor; siRNA, small interfering RNA; SSL, sterically stable liposomes.

Another approach to HSC-specific drug delivery has involved the coupling of peptide-modified proteins designed to recognise specific receptors on the target cells. These are generally considered easier to produce and to be less immunogenic, making them a more attractive drug targeting option ^30^. However, poor systemic stability associated with peptide-modified proteins is a potential hurdle that needs to be addressed ^30^. Initial studies successfully modified human serum albumin with multiple amino acid sequence Arg-Gly-Asp (RGD)-containing cyclic peptides to target collagen type VI receptor-expressing HSCs ^33^. Peptides containing RGD attachment sites that recognise collagen type VI receptor have since been successfully used in liver fibrosis models to deliver interferon-α1b-loaded liposomes to HSCs, with limited extra-hepatic uptake. Furthermore, targeted delivery of interferon-α1b significantly reduced liver fibrosis compared to non-targeted treatment ^34^. HSCs have also been successfully targeted through the generation of PDGFRβ-recognising peptides ^35^. As the amino acid sequences responsible for receptor binding are identical in rats and humans, this newly designed carrier may also have translational potential ^35^. This carrier has been used to deliver interferon-γ signalling peptide, resulting in marked inhibition of both early and established hepatic fibrosis without exhibiting any off-target effects ^36, 37^. Since its initial development, alterations to the PDGFRβ-recognising peptide delivery molecule have been made. Bansal and colleagues demonstrated that by switching from monocyclic binding peptides to bicyclic peptides, a more fitted interaction with the dimeric PDGFRβ is possible. This also reduced carrier size and complexity, making it potentially more feasible for clinical administration ^36^. Furthermore, addition of a PEG linker was used to prolong plasma half-life and stability as well as provide conformational flexibility for appropriate interaction with PDGFRβ ^36^.

A further approach to specific targeting of HSCs involves coupling liposomes to vitamin A, which is actively taken up by quiescent and activated HSCs ^38^. Sato *et al.* reported that siRNA-loaded liposomes could be targeted to HSCs using this approach and inhibited collagen deposition by myofibroblasts ^38^. Additionally, they report that activated HSCs, which reduce their vitamin A content during the activation process, take up vitamin A as effectively as resting HSCs ^38, 39^. A monoclonal human single-chain antibody fragment has also been generated that targets human HSCs via binding to the synaptophysin receptor ^40^. Follow-up studies revealed that the antibody specifically targets liver myofibroblasts, and not monocytes/macrophages during fibrosis, and was successfully used as a drug carrier ^41^. While the results are promising, there are a number of potential limitations associated with a monoclonal antibody drug carrier approach, including half-life, payload, and endocytotic potential ^30^.

Research has now also started to focus on the targeted delivery of therapeutic genes by adenovirus using the peptide-modified protein approach. Development of a fusion protein with affinity to adenovirus and PDGF peptide increased adenovirus gene transfer in isolated HSCs, whilst reducing tropism for hepatocytes ^42^. A more recent study has coupled adenovirus with a peptide showing affinity to the p75 neurotrophin receptor, which is present on HSCs and myofibroblasts ^43, 44^. Using this construct, the authors were able to show that *in vivo* expression of certain transcription factors enabled the re-programming of myofibroblasts to hepatocyte-like cells in fibrotic mouse livers and reduced liver fibrosis ^43^. Targeted delivery of transcription factors to myofibroblasts has also been achieved using adeno-associated virus vectors, which preferentially transduce myofibroblasts to generate hepatocytes that replicate function and proliferation of primary hepatocytes, and reduces liver fibrosis ^45^. The ability to specifically target scar-producing cells within the liver and reprogramme them to become cells with a positive functional benefit has massive therapeutic potential.

## Conclusion and future challenges

Recent advances in our basic understanding of hepatic myofibroblast biology have uncovered numerous potential therapeutic targets. Although it is likely that HSCs are a major contributor to the myofibroblast population, it is important that we continue to delve into the pool of cells giving rise to hepatic myofibroblasts, as further increasing our knowledge of the cellular and molecular mechanisms driving both hepatic fibrosis evolution and resolution will undoubtedly allow us to increase the potency and precision of new anti-fibrotic therapies. It also has to be borne in mind that most of the data regarding hepatic myofibroblast biology have been derived from pre-clinical animal models and there continues to be a significant lack of human data in this area. In the coming years, driven by the huge advances in ‘omics’ technologies, we should harness these powerful new methodologies to study human fibrotic liver tissue directly, to greatly increase our understanding of the cellular and molecular mechanisms driving human liver fibrosis, across the various aetiologies and stages of human chronic liver disease. The data accrued from these types of human studies can then be used to inform, guide, and focus our pre-clinical studies and increase the precision with which we can identify relevant, new anti-fibrotic therapeutic targets. Furthermore, given the heterogeneity of the myofibroblast response to different forms of liver injury, it is likely that future therapies will need to be tailored in a disease-specific manner. In the coming years, the identification of specific pro-scarring subpopulations within the liver’s mesenchymal cellular populations will hopefully facilitate and accelerate the discovery of effective, targeted anti-fibrotic therapies with the ultimate goal being the identification of specific markers that allow subpopulation-specific drug delivery to the scar-producing cells within the liver, with minimal effects on normal organ homeostasis. We are not there yet with regard to this degree of fidelity in treating patients with liver fibrosis; however, identification of organ-specific myofibroblast markers has the potential to open up a new era in precision medicine for liver fibrosis.

## Abbreviations

BSA, bovine serum albumin; ECM, extracellular matrix; EMEA, European Medicines Agency; FDA, US Food and Drug Administration; HSC, hepatic stellate cell; Lrat, lecithin-retinol acyltransferase; MMT, mesothelial to mesenchymal transition; PDGFRβ, platelet-derived growth factor receptor β; PF, portal fibroblast; RGD, Arg-Gly-Asp; siRNA, small interfering RNA.
